# The Effect of Calcium Sodium Phosphosilicate on Dentin Hypersensitivity: A Systematic Review and Meta-Analysis

**DOI:** 10.1371/journal.pone.0140176

**Published:** 2015-11-06

**Authors:** Mengjiao Zhu, Jialing Li, Bin Chen, Li Mei, Liang Yao, Jinhui Tian, Huang Li

**Affiliations:** 1 Department of Oral Sciences, Nanjing Stomatological Hospital, Medical School of Nanjing University, Nanjing, China; 2 Department of Orthodontics, Nanjing Stomatological Hospital, Medical School of Nanjing University, Nanjing, China; 3 Department of Periodontics, Nanjing Stomatological Hospital, Medical School of Nanjing University, Nanjing, China; 4 Discipline of Orthodontics, Department of Oral Science, Faculty of Dentistry, University of Otago, Dunedin, New Zealand; 5 Evidence-Based Medicine Center, School of Basic Medical Sciences, Lanzhou University, Gansu, China; University of Washington, UNITED STATES

## Abstract

**Objective:**

To investigate the effect of calcium sodium phosphosilicate (CSPS) in treating dentin hypersensitivity (DH) and to compare this effect to that of a negative (placebo) control.

**Materials and Methods:**

Several databases, including Medline, EMBASE, Web of Science, The Cochrane Library, and the Chinese Biomedical Literature Database, were searched to identify relevant articles published through January 2015; grey literature (i.e., academic literature that is not formally published) was also searched. Two authors performed data extraction independently and jointly using data collection forms. The primary outcome was the DH pain response to routine activities or to thermal, tactile, evaporative, or electrical stimuli, and the secondary outcome was the side effects of CSPS use. Each study was evaluated using the Cochrane Collaboration tool for assessing risk bias. Meta-analysis of studies with the same participant demographics, interventions, controls, assessment methods and follow-up periods was performed. The Grading of Recommendations Assessment Development and Evaluation System was used to assess the quality of the evidence and the risk of bias across studies.

**Results:**

Meta-analysis demonstrated that toothpaste containing 5% CSPS was more effective than the negative control at relieving dentin sensitivity, with the level of evidence classified as “moderate”. In addition, prophylaxis paste containing 15% calcium sodium phosphosilicate was favored over the negative control at reducing post-periodontal therapy hypersensitivity, with the level of evidence categorized as “low”. Only two studies reported side effects of CSPS use.

**Conclusions:**

The majority of studies found that calcium sodium phosphosilicate was more effective than the negative control at alleviating DH. Because strong evidence is scarce, high-quality, well-designed clinical trials are required in the future before definitive recommendations can be made.

## Introduction

Dentin hypersensitivity (DH) is defined as a short sharp pain that originates from exposed dentin in response to stimuli (typically thermal, evaporative, tactile, osmotic, or chemical) that cannot be ascribed to any other dental defect or pathology [1]. DH is a clinical oral health problem that affects the adult population, and its prevalence is high worldwide. Recent studies have demonstrated that 25%-46% of 18–70 years old people have this type of sensitivity [2–4].

The “hydrodynamic theory” states that the flow of dentinal fluid induced by perturbations within the dentinal tubules activates pulpal nociceptors and results in pain [5,6]. This convincing theory explains episodic and typical pain sensations. Based on this theory, the ideal DH treatment should either occlude the dentinal tubules or block neural transmission from the pulp [7]. Accordingly, several approaches (e.g., dentifrices containing potassium salts and in-office topical desensitizing agents) have been proposed for DH therapy, and at-home therapy is recommended as a preliminary treatment (e.g., desensitizing toothpaste) [8]. Potassium salts are known as nerve-numbing agents, and several clinical studies have shown that toothpaste containing potassium nitrate effectively reduces DH [9,10]. However, recent systematic reviews have failed to find sufficient evidence to support the efficacy of potassium nitrate toothpaste for DH [11,12]. Thus far, the therapeutic gold-standard treatment that predictably and completely eliminates DH has not been discovered [13].

A recent clinical study has indicated that calcium sodium phosphosilicate (CSPS) results in a greater reduction in sensitivity compared with potassium nitrate [14]. CSPS is a bioactive glass and, when exposed to body fluids, it reacts and deposits hydroxycarbonate apatite (HCA), a mineral chemically similar to that in enamel and dentin [15]. Early *in vitro* studies have demonstrated that CSPS forms a mineralized layer and occludes exposed dentin surfaces [16]. In addition, the layer formed by CSPS has been demonstrated to exhibit a greater reduction in permeability when challenged with citric acid compared with a control [17,18]. Recent clinical trials have also shown the efficiency of CSPS in reducing dentin sensitivity [19,20]. Over the past 10 years, CSPS has been included in the formulations of over 15 products, and these products are sold in over 20 countries [21]. NovaMin® (which is technically described as inorganic, amorphous CSPS), is the branded ingredient found in numerous professional and over-the-counter dental products designed to relieve tooth sensitivity.

Several authors have reviewed the literature in this area. A previously published systematic review found insufficient evidence to determine whether the effectiveness of CSPS was superior to that of a placebo [22]. Two additional systematic reviews found that CSPS might be effective in treating DH; however, the evidence was weak [23,24]. The only study that conducted quantitative analysis combined CSPSs of different concentrations together in meta-analysis, which might explain the high heterogeneity of the results [23]. Moreover, these previous reviews excluded all unpublished articles, which might result in publication bias [22–24].

Therefore, the current systematic review presents an overview of extant human clinical trials concerning the effect of using CSPS to treat DH compared with that of a negative (placebo) control.

## Materials and Methods

This study followed the Preferred Reporting Items for Systematic Reviews and Meta-Analyses (PRISMA) statement guidelines (www.prisma-statement.org). The review protocol is not available.

### Search Strategy

Five databases, namely Medline (via PubMed), EMBASE, Web of Science, CENTRAL (The Cochrane Library) and the Chinese Biomedical Literature Database, were searched on January 14, 2015. The search strategy aimed to identify all relevant articles published in English and Chinese (for details, see S1 Table). A supplemental manual search was conducted by reviewing the reference lists of the related papers and review articles. No restriction was applied regarding the date of publication.

The grey literature (i.e., academic literature that is not formally published) was also searched in ClinicalTrials.gov, the National Research Register, OpenGrey, and the World Health Organization’s International Clinical Trial Registry Platform.

### Study Selection

Two calibrated reviewers screened the titles and abstracts (when available) of the identified studies and extracted the data in duplicate. Any disagreement between the researchers was resolved via discussion, after consulting a third reviewer, until a consensus was reached. After a study was considered relevant, the full-text article was obtained and reviewed. The studies excluded during this or subsequent stages are listed in S2 Table, and the reasons for exclusion are noted. Only studies meeting the following criteria were included.

Participants: Humans with DH. Post-restorative hypersensitivity studies were excluded.

Intervention: CSPS-containing desensitizing formulations. Studies using topical CSPS in any modality, including a toothpaste, mouth rinse, and prophylaxis paste, were included. Moreover, there were no restrictions regarding the concentration, frequency, duration, or method of administration.

Comparison: Negative (placebo) control. (A negative control could be a vehicle containing the same formulation as the intervention vehicle but without the active ingredient or vehicle without desensitizing ingredients.)

Outcomes: The primary outcome was the DH pain response to routine activities or to thermal, tactile, evaporative, or electrical stimuli, and the secondary outcome was the side effects of CSPS use, including discomfort, oral hygiene deterioration, or dental staining.

Studies: Randomized controlled trials (RCTs).

### Data Extraction

The following data were extracted from each study:

- authors and publication year
- number and ages of participants
- interventions and control details
- follow-up period and check-time points
- assessment methods
- outcomes of interest

### Data Synthesis and Grading

Two reviewers independently completed a risk of bias assessment following the instructions of the Cochrane Handbook for Systematic Reviews of Interventions (Version 5.1.0. The Cochrane Collaboration; 2011. Available from: www.cochrane-handbook.org.) Disagreements were resolved via discussion, and a third reviewer was sought if necessary. The corresponding authors of the included studies were contacted as needed to locate unpublished material or to obtain missing data. The examiners assessed the presence of conflicts of interest by reviewing the authors’ disclosures and acknowledgments in the manuscript, based on the criterion used by Friedman and Richter [25].

The review authors defined four key domains for the Risk of Bias: random sequence generation, allocation concealment, blinding of participants and personnel, and blinding of outcome assessment. Once one or more key domains were judged as having high risks of bias, the risk was applied to the whole study.

Meta-analyses were planned only when sufficient similarities were found among the included studies with regard to the participant demographics, interventions, controls, assessment methods and follow-up periods. Subgroup analyses were conducted for different concentrations of CSPS or different methods of administration (e.g., toothpaste, mouth rinse, or prophylaxis paste). Mean differences (MDs) and standard deviations (SDs) were used to summarize data in studies with continuous outcomes. Heterogeneity was assessed using the I^2^ statistic [26]. Forest plots were constructed using Review Manager Version 5.2 (The Nordic Cochrane Centre, The Cochrane Collaboration, 2012). The Grading of Recommendations Assessment Development and Evaluation (GRADE) System’s Profiler 3.6 software [27] was used to assess the quality of the body of evidence with regard to the review question, as well as the risk of bias across studies.

## Results

### Study Selection

**Fig 1:**
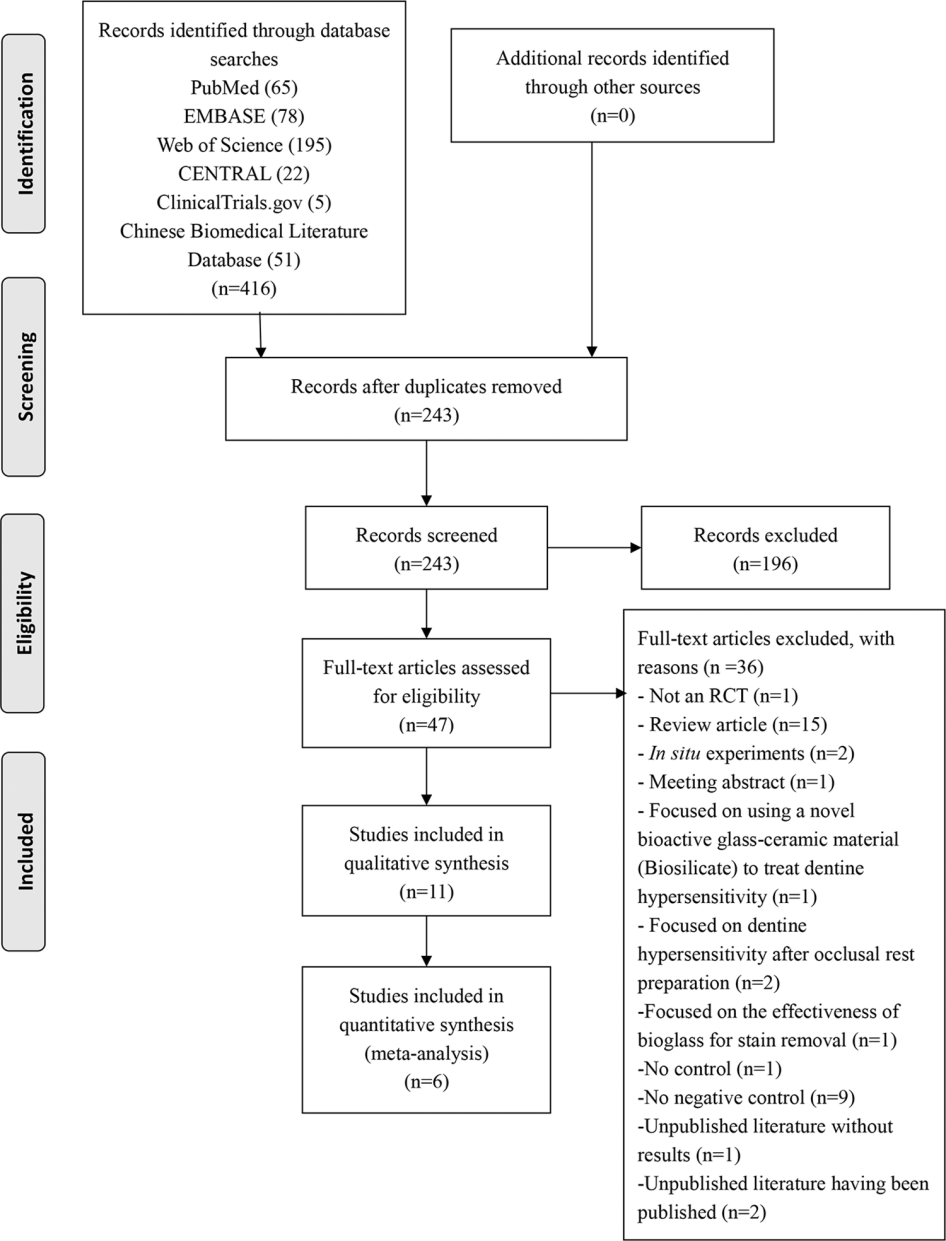
Systematic review flow diagram (RCT = Randomized controlled trials).

The initial search from all sources yielded 416 records. After screening the titles and abstracts, 369 records that were duplicated or unrelated to this systematic review were eliminated. As a result, 47 articles remained for full-text assessments; based on the predetermined eligibility criteria, 36 articles were excluded. A flowchart of the study selection process is shown in Fig 1. The remaining 11 reports were included in qualitative analysis [14,19,20,28–35].

### Descriptions of the Included Studies

Detailed data from the 11 included studies are listed in S3 Table. All included studies were RCTs comparing CSPS with a negative control. Four articles [19,31,33,35] that focused on post-periodontal therapy sensitivity were extracted as an independent analysis group.

CSPS was used in the form of self-administered toothpaste or professionally applied prophylaxis paste, with concentrations ranging from 2.5% [32] to 15% [19,33].

The follow-up times ranged from 15 days [28] to 8 weeks [29,32]. DH pain was elicited by tactile, evaporative, or thermal stimuli, and 2 studies [19,33] also reported self-assessed sensitivity. Different scales were used to quantify DH, and a 10-cm visual analogue scale (VAS) was the most commonly used for measurements [14,20,30,31,34].

### Primary Study Outcomes

Subjects in the DH group: Four studies [14,20,30,34] showed that toothpaste containing 5% CSPS was favored compared with a negative control at almost every time point; however, 1 study [30] did not report a significant difference between these treatments at 2 weeks. Furthermore, the results from the grey literature [28,29] did not show significant differences between the CSPS and control groups, and their results were not in agreement with the four abovementioned studies. Another study [32] observed effects of both 2.5% and 7.5% CSPS-containing toothpastes, showing that 7.5% CSPS was more effective at relieving DH than a negative control, whereas no significant difference was found between 2.5% CSPS and the negative control.

Subjects with post-periodontal therapy hypersensitivity: One study [35] showed that CSPS was better at relieving DH than a negative control over a period of less than 3 weeks; however, no significant difference was reported at 6 weeks. Another study [31] reported that 5% CSPS-containing toothpaste produced optimal results compared with a negative control. Two studies showed an advantage of a professionally applied prophylaxis paste containing 15% CSPS compared with a negative control with regard to preventing post-periodontal therapy hypersensitivity [19,33].

### Secondary Study Outcomes

Six studies did not observe adverse reactions during the study period [19,29–31,33,34], whereas another study [28] reported minor adverse events (e.g., gastrointestinal disorders, infections and infestations, injury, poisoning and procedural complications, and nervous system disorders). One study [32] observed adverse reactions to 2.5% and 7.5% CSPS, including soft tissue abnormalities that were not present at baseline in two participants in the control group, two in the 2.5% group and five in the 7.5% group. Approximately 57% of the participants reported at least one adverse event, although most were not orally related, and the event rate profiles of the three groups were similar. The remaining 3 studies [14,20,35] lacked information concerning adverse events.

### Risk of Bias

**Fig 2:**
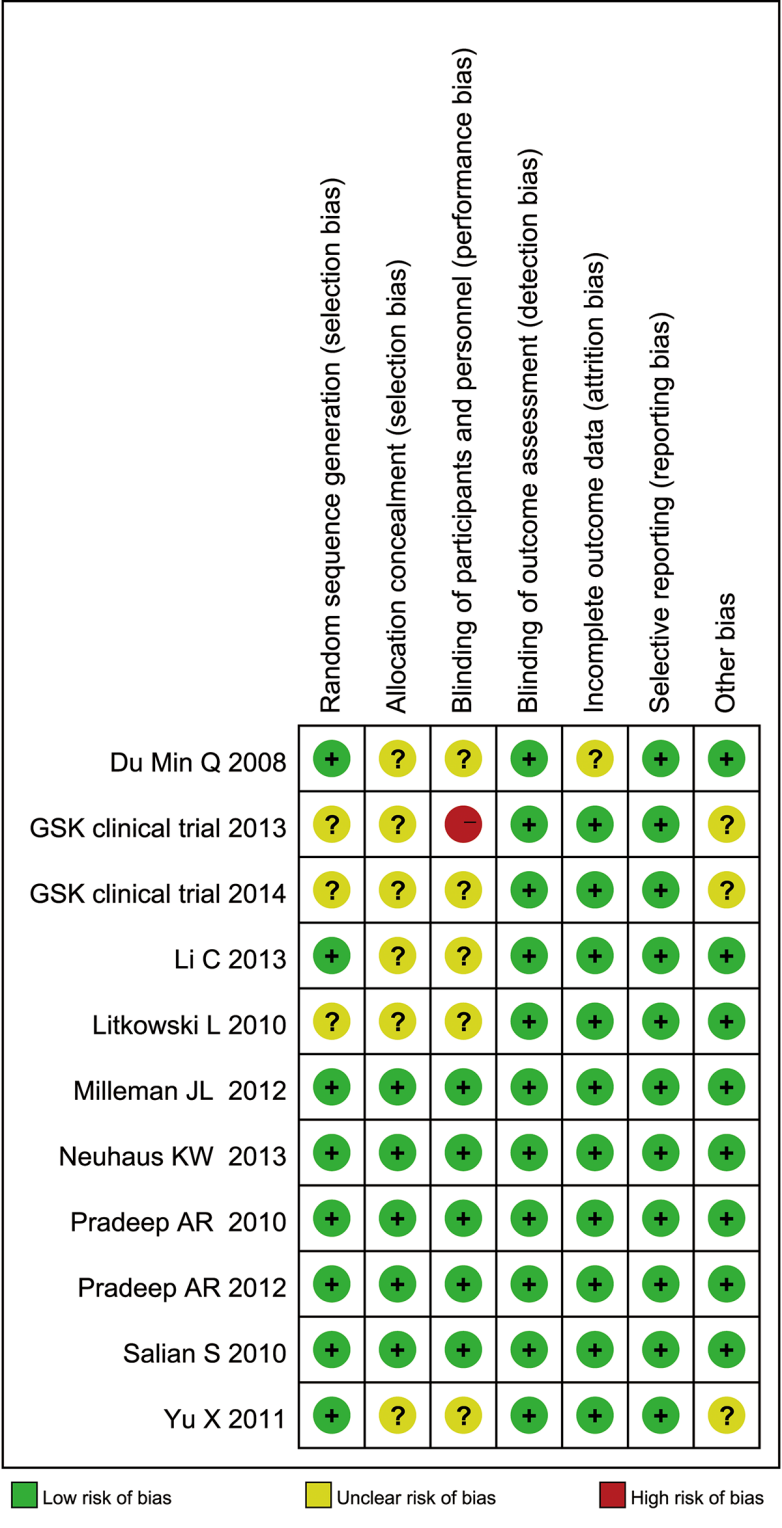
Risk of bias summary.

**Fig 3:**
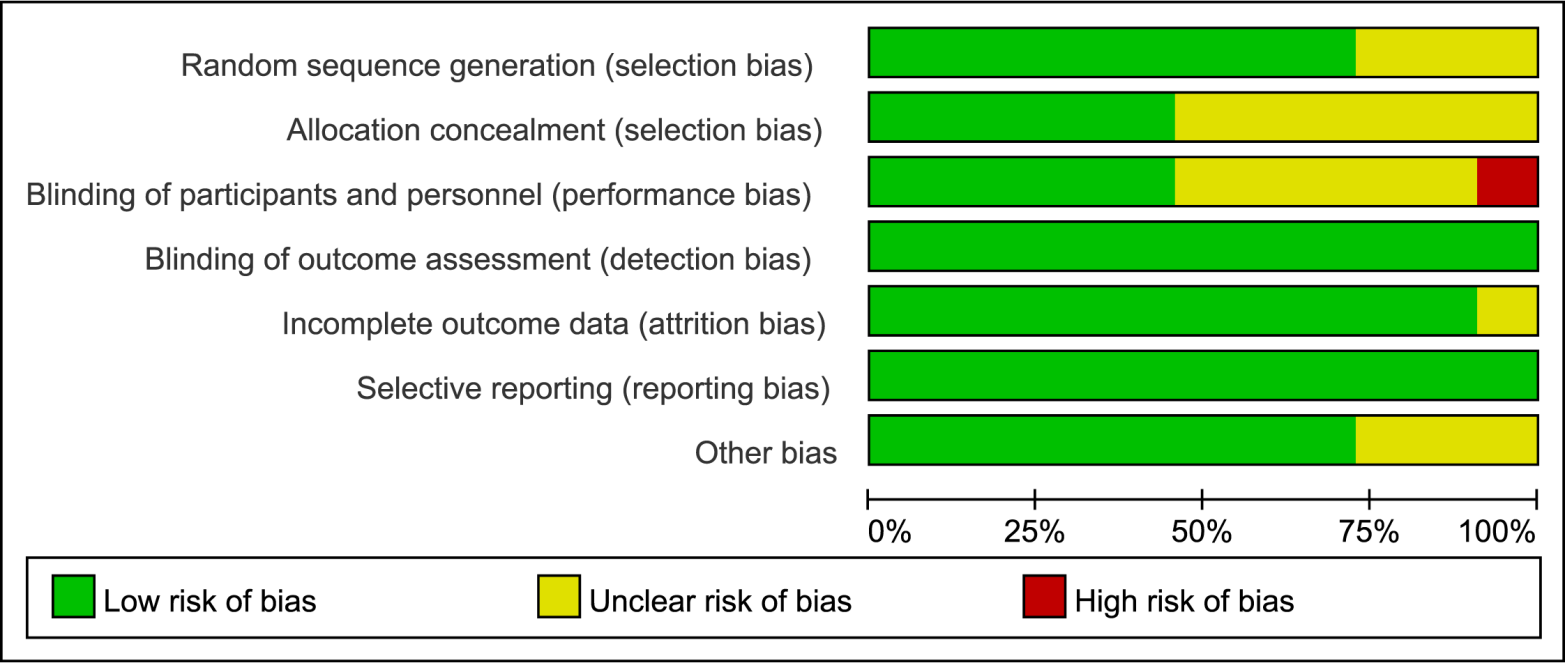
Risk of bias graph.

The risk of bias assessment revealed that 5 studies had a low risk of bias [14,19,20,33,34] and 1 had a high risk of bias [28]. The remaining 5 studies had an unclear risk of bias [29–32,35] (Figs 2 and 3). S4 Table summarizes the risks of bias across the studies.

Seven studies [19,28–30,32–34] were regarded as having potential conflicts of interests, either due to their own disclosure statements or to the review authors’ judgments.

### Result Synthesis and Evidence Grading

Meta-analysis was performed regarding the studies with the same types of participants, interventions, controls, assessment methods and follow-up periods. The study with a high risk of bias was excluded from meta-analysis [28]. The GRADE summary tables are shown in S4 Table.

#### 1. Toothpaste containing 5% CSPS versus negative control (Figs 4–7)

The 5% CSPS-containing toothpaste showed a better desensitizing effect at both 2 and 6 weeks regardless of the applied stimuli (evaporative, 2 weeks: MD = -0.68; 95% CIs = -1.15, -0. 20; I^2^ = 59%; evaporative, 6 weeks: MD = -1.69; 95% CIs = -1.86, -1.52; I^2^ = 42%; thermal, 2 weeks: MD = -0.59; 95% CIs = -1.33, 0.14; I^2^ = 84%; and thermal, 6 weeks: MD = -1.70; 95% CIs = -2.17, -1.23; I^2^ = 72%). The quality of evidence was categorized as “moderate”.

**Fig 4:**
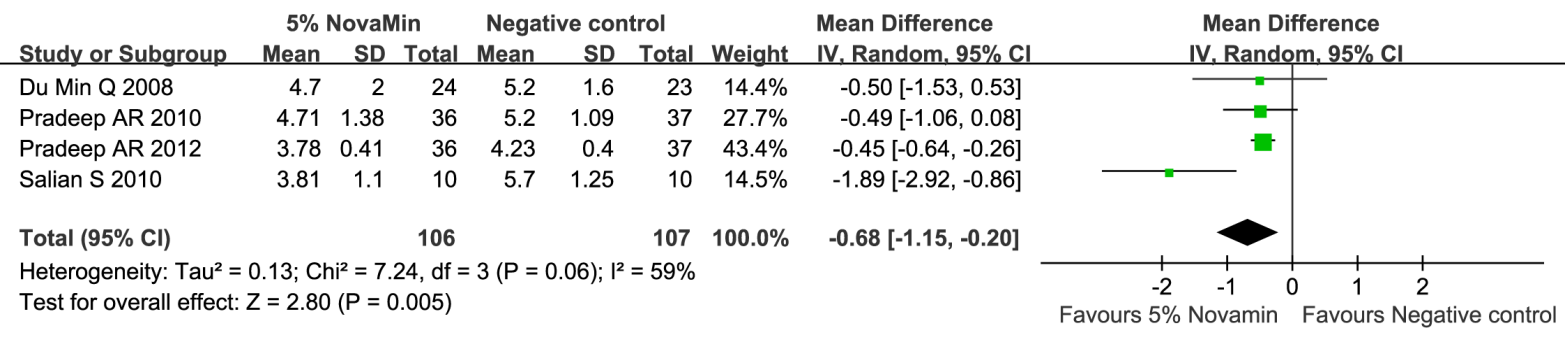
Forest plot comparing toothpaste containing 5% CSPS with a negative control for evaporative stimulus at 2 weeks.

**Fig 5:**
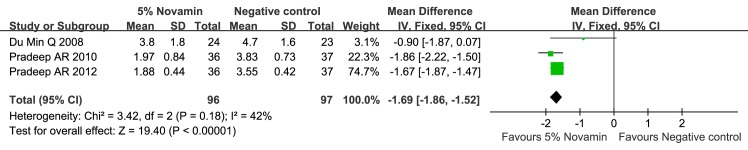
Forest plot comparing toothpaste containing 5% CSPS with a negative control for evaporative stimulus at 6 weeks.

**Fig 6:**
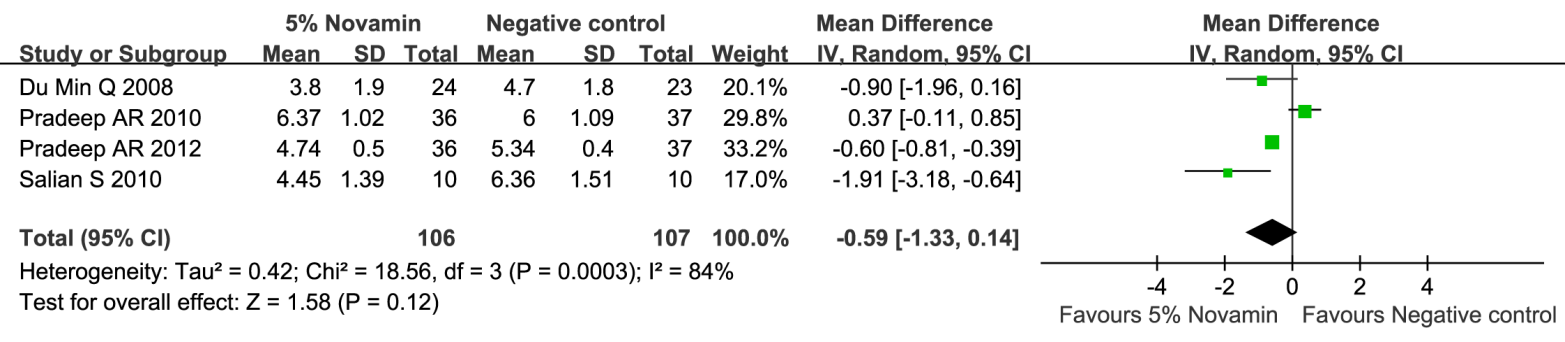
Forest plot comparing toothpaste containing 5% CSPS with a negative control for thermal stimulus at 2 weeks.

**Fig 7:**
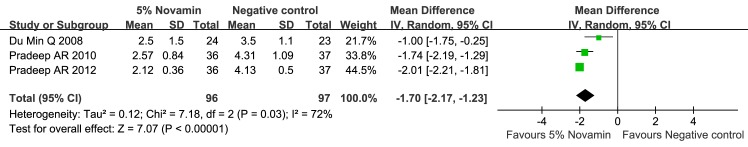
Forest plot comparing toothpaste containing 5% CSPS with a negative control for thermal stimulus at 6 weeks.

#### 2. Prophylaxis paste containing 15% CSPS versus negative control (Figs 8–11)

Prophylaxis paste containing 15% CSPS showed a better desensitizing effect on post-periodontal therapy DH pain than a negative control, immediately after prophylaxis and at 4 weeks, as determined using evaporative or tactile stimuli, and the results showed relatively low heterogeneity (evaporative, immediate: MD = -0.87; 95% CIs = -1.23, -0.51; I^2^ = 0%; evaporative, 4 weeks: MD = -0.93; 95% CIs = -1.11, -0.75; I^2^ = 41%; tactile, immediate: MD = -9.59; 95% CIs = -12.17, -7.01; I^2^ = 55%; and tactile, 4 weeks: MD = -8.34; 95% CIs = -10.87, -5.80; I^2^ = 0%). The quality of evidence was classified as “low”.

**Fig 8:**
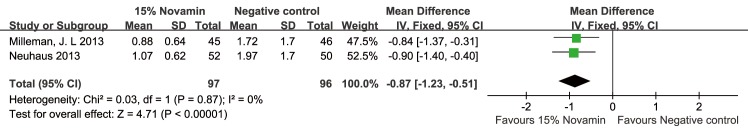
Forest plot comparing a prophylaxis paste containing 15% CSPS with a negative control for evaporative stimulus immediately after prophy.

**Fig 9:**
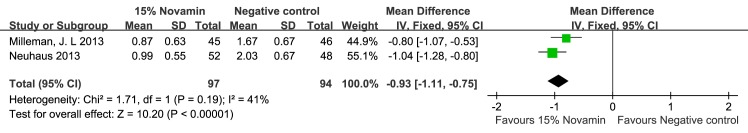
Forest plot comparing a prophylaxis paste containing 15% CSPS with a negative control for evaporative stimulus at 4 weeks.

**Fig 10:**
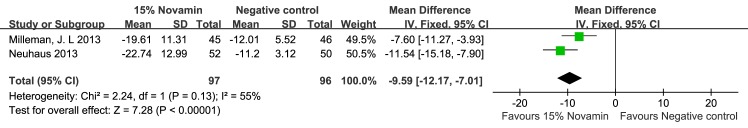
Forest plot comparing a prophylaxis paste containing 15% CSPS with a negative control for tactile stimulus immediately after prophy.

**Fig 11:**
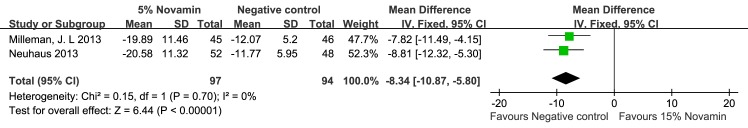
Forest plot comparing a prophylaxis paste containing 15% CSPS with a negative control for tactile stimulus at 4 weeks.

The two studies that assessed patients’ self-assessments of dentin sensitivity were not pooled because of clinical heterogeneity.

## Discussion

Based on the meta-analysis results, CSPS is more effective than negative controls in relieving DH, used either as a toothpaste to alleviate DH or as a prophylaxis paste to treat post-periodontal therapy DH.

However, this result was based on a small number of clinical trials (n = 11). In addition, seven of the included studies were industry-sponsored or partially industry-sponsored [19,28–30,32–34], and some authors were also employees of the companies [19,30,33]. There are a number of ways that industry sponsors can influence the outcome of a study. For example, sponsors might influence how the research question is framed, how the study is designed/conducted, and how the data are analyzed [36]. Industry-sponsored trials have been reported to be more likely to produce results that are favorable to the company sponsoring the research [37,38], and unfavorable results may not be published [39,40]. In this review, all trials included reported significant results in favor of CSPS, except for the grey literature [28]. Although publication bias was suspected, funnel plots could not be generated to recognize the extent of this bias due to the limited number of studies included in this review.

According to previous studies, periodontal therapy is a significant cause of dentin sensitivity. One reason is that dentinal tubules are exposed when the root cementum is removed during scaling procedures [41]. Thus, patients often report increased hypersensitivity following scaling and root planning [42]. In addition, periodontal treatment can result in an apical shift of the soft tissue margin with the recession of swollen gingiva, thereby increasing sensitivity [43]. Sensitivity after periodontal therapy differs from common DH in that it can peak during the first few days after scaling and root planning or periodontal surgery, and it is usually substantially reduced after the procedure [44]. Therefore, it is necessary to include participants who exhibit DH after periodontal therapy and to analyze them separately from patients who have not received periodontal therapy.

CSPS can be used as an intervention in the form of a homecare or in-office treatment. The concentration of CSPS for homecare use ranges from 2.5% to 7.5%. However, the concentration for professional use is usually higher. Self-administered dentifrice is used as an at-home treatment. It is simple and inexpensive to use and is the first choice of treatment for generalized DH affecting many teeth [8]. Alternatively, professionally applied prophylaxis paste is used as an in-office treatment. Its use is more complex, and it generally targets DH localized to one or more teeth [45].

The use of a control is one of the most controversial issues in trials of DH. A suitable negative control can be prepared by omitting the presumed active ingredient from the test-product formulation [1] (i.e., preparing the same vehicle without the claimed desensitizing ingredients). Another type of negative control frequently used in clinical trials is toothpaste without desensitizing ingredients as claimed by the authors. However, such toothpastes sometimes contain different fluoride salts, and studies have demonstrated that dentifrices containing fluoride can reduce DH [46,47]. Petersson has also reported that fluoride reduces and blocks fluid movement in dentin tubules by forming calcium–phosphorous precipitates, calcium fluoride (CaF_2_) and fluorapatite (FAp), thereby relieving DH [48]. However, high-fluoride products (e.g., varnishes) can occlude dentine tubules and provide relief from sensitivity [20], whereas low-fluoride products (e.g., dentifrices and mouthwashes) do not provide significant sensitivity relief. Thus, toothpastes containing fluoride should not be used as negative controls because it remains unclear whether or what concentration of fluoride reduces DH; however, toothpastes containing the same formulation as the intervention group (without the active ingredient) are appropriate for use. The appropriate positive control to use in these studies is also debatable. Compared with products that work by occluding the dentinal tubules, potassium salts produce a desensitizing effect by increasing the concentration of extracellular potassium around nerve fibers, blocking the passage of nerve stimuli [49]. The United States Food and Drug Administration has recognized potassium nitrate as a safe and effective treatment for DH [50]. Studies have also demonstrated that DH is relieved with the use of toothpaste containing 5% potassium nitrate [9,51]. However, *in vitro* experiments have indicated that potassium-induced effects are transient and reversible [52]. In addition, a recent systematic Cochrane review has failed to obtain strong evidence supporting the use of potassium-containing toothpastes to treat DH [12]. Because a well-established gold standard product for treating DH is not available, the need for a positive control remains controversial.

Tactile, thermal and evaporative air stimuli are recommended for eliciting DH pain because they are physiological and controllable variables. In addition, because DH responses can differ for different stimuli, at least 2 stimuli should be used [1]. All of the studies included in this meta-analysis used 2 or more of the aforementioned stimuli, and 2 studies also used self-assessed sensitivity to “everyday” stimuli, which is subjective and difficult to assess but might be essential from the patient’s perspective [1]. Furthermore, the least severe stimulus should be applied first to prevent interpretation error, and a 5-minute interval should be employed between stimuli to minimize their interaction [14].

Because pain is an inherently subjective symptom, an objective assessment method is not available. Studies have reported numerous pain assessment scales, including (but not limited to) the VAS, Numeric Rating Scale (NRS), and Verbal Rating Scale (VRS) [53]. The VAS is the most frequently used scale, for which the patient marks his or her pain, ranging from no pain to intolerable pain, on a 10-cm line. Certain researchers prefer to use the NRS [1], which is a segmented, numeric version of the VAS. In fact, a systematic review has indicated that the NRS is associated with increased compliance compared with the other scales. Although the VAS offers greater measurement sensitivity, it might be more complicated to use, and it is associated with higher error rates, particularly among elderly users [54]. The VRS uses words via a scaling technique to determine variations in pain; however, it might not have an optimal discriminatory capacity, its category scales are discontinuous, and its average responses are not always meaningful and can be misleading [1,54]. Although no gold standard has been established to assess DH pain, the VAS is recommended for relatively younger patients with better compliance, whereas the NRS is recommended for relatively older patients.

The duration of DH clinical trials depends on whether they evaluate the short- or long-term effects of a product. In general, the trial duration should be sufficient to produce the maximum efficacy of the active agent while minimizing the magnitudes of any placebo effects; however, the optimum time courses for different agents depend on their modes of action. Although a previous review has suggested that 8 weeks is a suitable duration for most DH clinical trials [1], distinguishing the original DH episode from a recent occurrence becomes more difficult as the duration increases. According to the included clinical trials, relief from pain associated with the use of CSPS toothpaste is likely cumulative with continued use; thus, a follow-up period of no less than 4 weeks is usually recommended to obtain significant CSPS results compared with placebo or a positive control.

Only one study reported adverse effects associated with CSPS, showing increased efficacy but a higher incidence of gingival inflammation with increasing concentrations of CSPS [32]. However, the number of participants in each treatment group was small (n = 22); thus, it is necessary to interpret the safety data with caution. The authors reported that gingival inflammation might be explained by the use of a new toothbrush dispensed at every visit or by the ability of CSPS to release calcium and phosphate into the aqueous environment, resulting in an elevated pH. Conversely, evidence acquired from a 6-week study has also shown that 5% CSPS-containing dentifrice significantly improves oral health [55]. Therefore, whether higher concentrations of CSPS have more adverse/beneficial effects remains unclear, and additional clinical trials with larger sample sizes are required to determine the optimal concentration of CSPS.

Finally, this systematic review has several limitations. First, selection bias may have occurred because the search was restricted to publications in English and Chinese. Second, some of the included studies did not report how randomization was applied or whether treatment allocations were blinded to caregivers [28–32,35]. Finally, all of the comparisons performed included a small number of studies, which may have contributed to a low power for meta-analyses.

## Conclusions

Within the limitations of this systematic review, the evidence suggests that 5% CSPS-containing toothpaste is effective for use as an at-home treatment to relieve DH. Prophylaxis paste containing 15% CSPS is also favored over a negative control at reducing post-periodontal therapy hypersensitivity. The levels of evidence for these findings are classified as “moderate” and “low”, respectively. In addition, whether high CSPS concentrations (i.e., more than 5%) have more side effects remains unclear; however, high concentrations should be used with caution in products for home use. In the future, more high-quality, non-industry-supported clinical studies in this area should be conducted before any definitive recommendations can be made.

## Supporting Information

S1 PRISMA Checklist (DOCX)

S1 Table Search strategy. (DOCX)

S2 Table Articles excluded from this review. (DOCX)

S3 Table Summary of the Included Studies. (DOCX)

S4 Table GRADE Profile Table. (DOCX)

S5 Table Risk of bias in included studies. (DOCX)
