# Supplementary material for: The Effect of Calcium Sodium Phosphosilicate on Dentin Hypersensitivity: A Systematic Review and Meta-Analysis
Source: PLoS One. 2015 Nov 6;10(11):e0140176. doi: 10.1371/journal.pone.0140176 (PMC4636152; doi:10.1371/journal.pone.0140176)
Supplement: S1 Table — (DOCX) [file pone.0140176.s002.docx]

**Table A. Search strategy for Medline via PubMed**

| #1 | “dentin sensitivity” [MeSH] |
| --- | --- |
| #2 | “dentin sensitivity” OR “dentin* sensitivity” [Title/Abstract] |
| #3 | “dentin hypersensitivity” OR “dentin* hypersensitivity” [Title/Abstract] |
| #4 | sensitivity OR hypersensitivity OR desensiti* [Title/Abstract] |
| #5 | #1 or #2 or #3 or #4 |
| #6 | bioglass OR bio-glass OR “bioactive glass” OR 45S5 OR Novamin OR “calcium sodium phosphosilicate” [Title/Abstract] |
| #7 | #5 AND #6 |

**Table B. Search strategy for EMBASE**

| #1 | ‘dentin sensitivity’/exp OR ‘dentin sensitivity’ |
| --- | --- |
| #2 | ‘dentin hypersensitivity’ |
| #3 | dentin* AND sensitivity |
| #4 | dentin* AND hypersensitivity |
| #5 | sensitivity OR hypersensitivity OR desensiti* |
| #6 | #1 OR #2 OR #3 OR #4 OR #5 |
| #7 | bioglass OR bio-glass OR ‘bioactive glass’ OR 45S5 OR Novamin OR ‘calcium sodium phosphosilicate’ |
| #8 | #6 AND #7 |

**Table C. Search strategy for Web of Science**

| #1 | Topic: (dentin sensitivity) OR Topic: (dentin* sensitivity) |
| --- | --- |
| #2 | Topic: (dentin hypersensitivity) OR Topic: (dentin* hypersensitivity) |
| #3 | Topic: (sensitivity) OR Topic: (hypersensitivity) OR Topic: (densensiti*) |
| #4 | #1 or #2 or #3 |
| #5 | Topic: (bioglass) OR Topic: (bio-glass) OR Topic: (bioactive glass) OR Topic: (45S5) OR Topic: (Novamin) OR Topic: (calcium sodium phosphosilicate) |
| #6 | #4 AND #5 |

**Table D. Search strategy for CENTRAL(The Cochrane Library)**

| #1 | MESH descriptor: [Dentin Sensitivity] explode all trees |
| --- | --- |
| #2 | “dentin sensitivity”:ti,ab,kw or “dentin* sensitivity”:ti,ab,kw or “dentin hypersensitivity”:ti,ab,kw or “dentin* hypersensitivity”:ti,ab,kw (Word variations have been searched) |
| #3 | “sensitivity”:ti,ab,kw or “hypersensitivity”:ti,ab,kw or densensiti*:ti,ab,kw (Word variations have been searched) |
| #4 | #1 or #2 or #3 |
| #5 | “bioglass”:ti,ab,kw or bio-glass:ti,ab,kw or “bioactive glass”:ti,ab,kw or 45S5:ti,ab,kw or Novamin:ti,ab,kw (Word variations have been searched) |
| #6 | “calcium sodium phosphosilicate” (Word variations have been searched) |
| #7 | #5 or #6 |
| #8 | #4 and #7 |

**Table E. Search strategy for ClinicalTrials.gov**

| #1 | “dentin sensitivity” OR “dentin hypersensitivity” |
| --- | --- |
| #2 | sensitivity OR hypersensitivity OR desensiti* |
| #3 | #1 OR #2 |
| #4 | bioglass OR bio-glass OR “bioactive glass” OR 45S5 OR Novamin OR “calcium sodium phosphosilicate” |
| #5 | #3 AND #4 |

**Table F. Search strategy for Chinese Biomedical Literature Database**

| #1 | “牙本质过敏” [不加权：扩展] |
| --- | --- |
| #2 | 牙本质过敏 OR 敏感 OR 过敏 OR 脱敏 OR 超敏 |
| #3 | #1 OR #2 |
| #4 | 生物玻璃 OR 生物活性玻璃 OR 生物活性材料 OR 诺华敏OR 舒适达 OR bioglass OR bio-glass OR “bioactive glass” OR 45S5 OR Novamin OR “calcium sodium phosphosilicate” |
| #5 | 硅 AND 磷 AND 钙 |
| #6 | Si AND P AND Ca |
| #7 | #4 OR #5 OR #6 |
| #8 | #3 AND #7 |
