# Supplementary material for: The Effect of Calcium Sodium Phosphosilicate on Dentin Hypersensitivity: A Systematic Review and Meta-Analysis
Source: PLoS One. 2015 Nov 6;10(11):e0140176. doi: 10.1371/journal.pone.0140176 (PMC4636152; doi:10.1371/journal.pone.0140176)
Supplement: S2 Table — (DOCX) [file pone.0140176.s003.docx]

**S2 Table. Articles excluded from this review**

| Article | Reason for exclusion |
| --- | --- |
| West NX, Macdonald EL, Jones SB, Claydon NC, Hughes N, Jeffery P. Randomized in situ clinical study comparing the ability of two new desensitizing toothpaste technologies to occlude patent dentin tubules. J Clin Dent. 2011;22: 82-89. | *In situ* design |
| Amaechi BT, Mathews SM, Mensinkai PK. Effect of theobromine-containing toothpaste on dentin tubule occlusion in situ. Clin Oral Investig. 2015;19: 109-116. | *In situ* design |
| Gillam DG, Weiss G, Bulman JS, Newman H. Efficacy of a novel Bioglass® dentifrice: results of an 8-week study [abstract]. Int Dent J. 2000;50: 355 | Meeting abstract |
| Rajesh KS, Hedge S, Arun Kumar MS, Shetty DG. Evaluation of the efficacy of a 5% calcium sodium phosphosilicate (Novamin) containing dentifrice for the relief of dentinal hypersensitivity: a clinical study. Indian J Dent Res. 2012;23: 363-367. | Not an RCT |
| Tirapelli C, Panzeri H, Lara EH, Soares RG, Peitl O, Zanotto ED. The effect of a novel crystallised bioactive glass-ceramic powder on dentine hypersensitivity: A long-term clinical study. J Oral Rehabil. 2011;38: 253-262. | Focused on the use of a novel  bioactive glass-  ceramic material (Biosilicate)  to treat dentine hypersensitivity |
| Banerjee A, Hajatdoost-Sani M, Farrell S, Thompson I. A clinical evaluation and comparison of bioactive glass and sodium bicarbonate air-polishing powders. J Dent. 2010;38: 475-479. | Focused on the effective-  ness of bioglass for stain removal |
| Wefel JS. NovaMin: likely clinical success. Adv Dent Res. 2009;21: 40-43. | Review article |
| Thomas MS. Dentin hypersensitivity. J Am Dent Assoc. 2011;142: 16. | Review article |
| Talioti E, Hill R, Gillam DG. The Efficacy of Selected Desensitizing OTC Products: A Systematic Review. ISRN Dent. 2014;2014: 865761. | Review article |
| Sohrabi K, Saraiya V, Laage TA, Harris M, Blieden M, Karimbux N. An evaluation of bioactive glass in the treatment of periodontal defects: a meta-analysis of randomized controlled clinical trials. J Periodontol. 2012;83: 453-464. | Review article |
| Shiau HJ. Dentin Hypersensitivity. J Evid Based Dent Pract. 2012;12(S3): 220-228. | Review article |
| Pillai VP, Neelakantan P. Desensitizing toothpastes for treatment of dentin hypersensitivity. Int J PharmTech Res. 2013;5:1769-1773. | Review article |
| Miglani S, Aggarwal V, Ahuja B. Dentin hypersensitivity: Recent trends in management. J Conserv Dent. 2010;13: 218-224. | Review article |
| Layer TM. Development of a fluoridated, daily-use toothpaste containing NovaMin technology for the treatment of dentin hypersensitivity. J Clin Dent. 2011;22: 59-61. | Review article |
| Hench LL, Greenspan D. Interactions between Bioactive Glass and Collagen: A Review and New Perspectives. J Aust Ceram Soc. 2013;49: 1-40. | Review article |
| Greenspan DC. NovaMin and tooth sensitivity--an overview. J Clin Dent. 2010;21: 61-65. | Review article |
| Gendreau L, Barlow AP, Mason SC. Overview of the clinical evidence for the use of NovaMin in providing relief from the pain of dentin hypersensitivity. J Clin Dent. 2011;22: 90-95. | Review article |
| Burwell AK, Litkowski LJ, Greenspan DC. Calcium sodium phosphosilicate (NovaMin): remineralization potential. Adv Dent Res. 2009;21: 35-39. | Review article |
| Ali S, Farooq I, Iqbal K. A review of the effect of various ions on the properties and the clinical applications of novel bioactive glasses in medicine and dentistry. Saudi Dent J. 2014;26: 1-5. | Review article |
| Patsouri A, Mavrogiannea A, Pepelassi E, Gaintantzopoulou M, Kakaboura A. Clinical effectiveness of a desensitizing system on dentin hypersensitivity in periodontitis patients. Am J Dent. 2011;24: 85-92. | Inclusion criterion for control not met |
| Acharya AB, Surve SM, Thakur SL. A clinical study of the effect of calcium sodium phosphosilicate on dentin hypersensitivity. J Clin Exp Dent. 2013;5: e18-22. | Inclusion criterion for control not met |
| Li X, Yang H, Chen N. The clinical effectiveness of a calcium sodium phosphosilicate desensitizer in reducing root-dentin hypersensitivity in the elderly. Chin J Geriatr. 2013;32:430-432. | Inclusion criterion for control not met |
| Narongdej T, Sakoolnamarka R, Boonroung T. The effectiveness of a calcium sodium phosphosilicate desensitizer in reducing cervical dentin hypersensitivity: a pilot study. J Am Dent Assoc. 2010;141: 995-999. | Inclusion criterion for control not met |
| Satyapal T, Mali R, Mali A, Patil V. Comparative evaluation of a dentifrice containing calcium sodium phosphosilicate to a dentifrice containing potassium nitrate for dentinal hypersensitivity: A clinical study. J India Soc Periodontol. 2014;18: 581-585. | Inclusion criterion for control not met |
| Sharma N, Roy S, Kakar A, Greenspan DC, Scott R. A clinical study comparing oral formulations containing 7.5% calcium sodium phosphosilicate (NovaMin), 5% potassium nitrate, and 0.4% stannous fluoride for the management of dentin hypersensitivity. J Clin Dent. 2010;21: 88-92. | Inclusion criterion for control not met |
| 宋子元, 谢咏梅, 许乃同, 王潇潇, 钟晓丽. 四种方法治疗牙本质敏感症的疗效比较. 北京口腔医学. 2012;20:222-224. | Inclusion criterion for control not met |
| Surve SM, Acharya AB, Shetty A, Thakur SL. Efficacy of calcium sodium phosphosilicate in managing dentinal hypersensitivity. Gen Dent. 2012;60: e308-311. | Inclusion criterion for control not met |
| Bm S, P P, Sanghani NN. Chair Side Application of NovaMin for the Treatment of Dentinal Hypersensitivity- A Novel Technique. J Clin Diagn Res. 2014;8: ZCO05-08. | Inclusion criterion for control not met |
| Patel PV, Patel A, Kumar S, Holmes JC. Evaluation of ozonated olive oil with or without adjunctive application of calcium sodium phosphosilicate on post-surgical root dentin hypersensitivity: a randomized, double-blinded, controlled, clinical trial. Minerva Stomatol. 2013;62: 147-161. | Inclusion criterion for control not met |
| 闫明, 李培, 李亮, 杨成, 刘克礼, 夏琳. 三种脱敏剂治疗(牙合)支托窝牙本质敏感症的疗效比较. 口腔材料器械杂志. 2014;23: 53-56. | Focused on dentine  hypersensitivity after  occlusal rest preparation |
| 艾连弟, 闫明, 李培, 李亮, 杨成, 徐军义. 三种牙本质脱敏剂治疗(牙合)支托窝牙本质敏感效果分析. 广东牙病防治. 2014;22: 82-85. | Focused on dentine  hypersensitivity after  occlusal rest preparation |
