# Supplementary material for: The Effect of Calcium Sodium Phosphosilicate on Dentin Hypersensitivity: A Systematic Review and Meta-Analysis
Source: PLoS One. 2015 Nov 6;10(11):e0140176. doi: 10.1371/journal.pone.0140176 (PMC4636152; doi:10.1371/journal.pone.0140176)
Supplement: S3 Table — (DOCX) [file pone.0140176.s004.docx]

**S3 Table. Summary of the Included Studies**

| **Type of Participants** | **First author (year) [reference number]** | **N of Observations by Group** | **Age** | **Follow-up protocol** | **Type of Intervention** | **Comparator/control** | **Assessment method** | **Primary outcome** |
| --- | --- | --- | --- | --- | --- | --- | --- | --- |
|  |  |  |  |  |  |  |  |  |
|  |  |  |  |  |  |  |  |  |
| **People with DH** | Du Min Q (2008) [30] | 71 participants | 21-56 yrs | Baseline, 2 w, 6 w | Toothpaste containing 5% CSPS, Twice a day | The same toothpaste without CSPS | Evaporative, thermal, VAS (0-10 cm) ≥4 | Test/control **(**mean±SD#**)** |
|  |  |  |  |  |  |  |  | Evaporative |
|  |  |  |  |  |  |  |  | Baseline: 5.9±1.5/6.1±1.3 |
|  |  |  |  |  |  |  |  | 2 w: 4.7±2.0/5.2±1.6 |
|  |  |  |  |  |  |  |  | 6 w: 3.8±1.8*/4.7±1.6 |
|  |  |  |  |  |  |  |  | Thermal |
|  |  |  |  |  |  |  |  | Baseline: 4.0±2.3/4.6±1.4 |
|  |  |  |  |  |  |  |  | 2 w: 3.8±1.9*/4.7±1.8 |
|  |  |  |  |  |  |  |  | 6 w: 2.5±1.5*/3.5±1.1 |
|  | Litkowski L (2010) [32] | 66 participants | Mean 40.6/39.2/36.7 yrs | Baseline, 2 w, 4 w, 8 w | Toothpaste containing 2.5% CSPS, Toothpaste containing 7.5% CSPS, Twice a day | Toothpaste without CSPS | Tactile, thermal, 30≤VAS (0-100 mm) ≤70 | Test1/test2/control **(**mean±SD^¶^**)** |
|  |  |  |  |  |  |  |  | Tactile |
|  |  |  |  |  |  |  |  | Baseline: 48.4±10.32/49.2±10.32/47.5±10.79 |
|  |  |  |  |  |  |  |  | 2 w: 34.8±17.33/28.1±13.95*/42.4±11.74 |
|  |  |  |  |  |  |  |  | 4 w: 29.8±16.78/21.7±13.37*/33.8±12.99 |
|  |  |  |  |  |  |  |  | 8 w: 30.8±23.64/13.2±10.24*/31±11.10 |
|  |  |  |  |  |  |  |  | Thermal |
|  |  |  |  |  |  |  |  | Baseline: 50.0±7.97/49.4±8.44/48.7±8.91 |
|  |  |  |  |  |  |  |  | 2 w: 44.9±16.46/36.3±10.66*/46.1±4.2 |
|  |  |  |  |  |  |  |  | 4 w: 35.5±19.04/27.9±11.84*/41.9±11.49 |
|  |  |  |  |  |  |  |  | 8 w: 34.5±20.55/22±12.97*/34.2±14.34 |
|  | Pradeep AR (2010) [14] | 110 participants | 20-60 yrs (mean 41.9/39.4 yrs) | Baseline, 2 w, 6 w | Toothpaste containing 5% CSPS, Twice a day | The same toothpaste without CSPS | Evaporative, thermal, VAS (0-10 cm) ≥4 | Test/control (mean±SD^¶^) |
|  |  |  |  |  |  |  |  | Evaporative |
|  |  |  |  |  |  |  |  | Baseline: 7.17±1.5/6.40±1.09 |
|  |  |  |  |  |  |  |  | 2 w: 4.71±1.38*/5.20±1.09 |
|  |  |  |  |  |  |  |  | 6 w: 1.97±0.84*/3.83±0.73 |
|  |  |  |  |  |  |  |  | Thermal |
|  |  |  |  |  |  |  |  | Baseline: 8.43±1.26/6.91±1.28 |
|  |  |  |  |  |  |  |  | 2 w: 6.37±1.02*/6.00±1.09 |
|  |  |  |  |  |  |  |  | 6 w: 2.57±0.84*/4.31±1.09 |
|  | Salian S (2010) [34] | 30 participants | 20-50 yrs (mean 39.4/38.2 yrs) | Baseline, 2 w, 4 w | Toothpaste containing 5% CSPS, Twice a day | Dentifrice without desensitizing ingredients | Tactile, evaporative, thermal VAS (0-10 cm) ≥4 | Test/control (mean±SD) |
|  |  |  |  |  |  |  |  | Tactile |
|  |  |  |  |  |  |  |  | Baseline: 3.96±0.80/3.55±0.81 |
|  |  |  |  |  |  |  |  | 2 w: 2.55±0.80*/3.55±0.81 |
|  |  |  |  |  |  |  |  | 4 w: 0.96±0.81*/3.55±0.79 |
|  |  |  |  |  |  |  |  | Evaporative |
|  |  |  |  |  |  |  |  | Baseline: 5.77±1.02/5.67±1.23 |
|  |  |  |  |  |  |  |  | 2 w: 3.81±1.10*/5.70±1.25 |
|  |  |  |  |  |  |  |  | 4 w: 1.99±1.22*/5.41±1.29 |
|  |  |  |  |  |  |  |  | Thermal |
|  |  |  |  |  |  |  |  | Baseline: 6.85±1.14/6.36±1.51 |
|  |  |  |  |  |  |  |  | 2 w: 4.45±1.39*/6.36±1.51 |
|  |  |  |  |  |  |  |  | 4 w: 2.37±1.20*/5.97±1.52 |
|  | Pradeep AR (2012) [20] | 149 participants | 20-60 yrs (mean 38.9/40.6 yrs) | Baseline, 2 w, 6 w | Toothpaste containing 5% CSPS, Twice a day | Placebo toothpaste | Evaporative, thermal, VAS (0-10 cm) ≥4 | Test/control **(**mean±SD**)** |
|  |  |  |  |  |  |  |  | Evaporative |
|  |  |  |  |  |  |  |  | Baseline: 5.55±0.34/5.31±0.46 |
|  |  |  |  |  |  |  |  | 2 w: 3.78±0.41*/4.23±0.40 |
|  |  |  |  |  |  |  |  | 6 w: 1.88±0.44*/3.55±0.42 |
|  |  |  |  |  |  |  |  | Thermal: |
|  |  |  |  |  |  |  |  | Baseline: 6.95±0.42/6.29±0.41 |
|  |  |  |  |  |  |  |  | 2 w: 4.74±0.50*/5.34±0.40 |
|  |  |  |  |  |  |  |  | 6 w: 2.12±0.36*/4.13±0.50 |
|  | GSK clinical trial (2013) [28] | 194 participants | 18-50 yrs (mean 37.63/38.3/36.61) | Baseline, 3 d, 7 d, 10 d, 15 d | Toothpaste containing 7.5% CSPS, Toothpaste containing 5% CSPS, Twice a day | CSPS-free placebo toothpaste | Evaporative, thermal, VAS (0-100 mm) >25 | Test1/test2/control (adjusted mean change from baseline [95% CIs]) |
|  |  |  |  |  |  |  |  | Evaporative |
|  |  |  |  |  |  |  |  | 7 d: -16.4(-19.8, -12.93)/-17.4(-20.75, -14.10)/-16.4(-19.78, -13.02) |
|  |  |  |  |  |  |  |  | 15 d: -26.1(-29.4, -22.72)/-28.4(-31.60, -25.12)/-26.2(-29.54, -22.95) |
|  |  |  |  |  |  |  |  | Thermal |
|  |  |  |  |  |  |  |  | 7 d: -21.5(-26.14, -16.81)/-22.5(-26.99, -17.93)/-21.8(-26.44, -17.21) |
|  |  |  |  |  |  |  |  | 15 d: -32.8(-37.55, -28.00)/-32.2(-36.86, -27.59)/-32.3(-37.04, -27.60) |
|  | GSK clinical trial (2014) [29] | 134 participants | 18-55 yrs (mean 35.4/33.2) | Baseline, 4 w, 8 w | Toothpaste containing 5.0% CSPS and 1500 ppmF as sodium monofluorophosphate | Toothpaste containing 0% CSPS and 1500 ppmF as sodium monofluorophosphate | Evaporative, 4-point Schiff-score (0, 1, 2, 3)≥2 & 10-point visual analog scale (1-10), Tactile (gram) | Test/control 1 (change from baseline) |
|  |  |  |  |  |  |  |  | Evaporative (VAS[0, 1, 2, 3] [mean{95% CIs}]) |
|  |  |  |  |  |  |  |  | 4 w: -0.36(-0.53, -0.20)/-0.45(-0.62, -0.28) |
|  |  |  |  |  |  |  |  | 8 w: -0.55(-0.78, -0.33)*/-0.92(-1.15, -0.68) |
|  |  |  |  |  |  |  |  | Tactile (mean±SD) |
|  |  |  |  |  |  |  |  | 4 w: 7.31±11.227*/3.64±6.030 |
|  |  |  |  |  |  |  |  | 8 w: 10.15±15.100/11.97±16.486 |
|  |  |  |  |  |  |  |  | Evaporative (VAS[1-10] [mean{95% CIs}]) |
|  |  |  |  |  |  |  |  | 4 w: -1.22(-1.76, -0.69)/-1.25(-1.80, -0.71) |
|  |  |  |  |  |  |  |  | 8 w: -1.76(-2.35, -1.17)/-2.06(-2.67, -1.46) |
| **People with post-periodontal therapy hypersensitivity** | Yu X(2011) [35] | 60 participants | 22-57 yrs (mean 42.3 yrs) | Baseline, 1 w, 2 w, 3 w, 6 w | Bio-glass powder and 7% bio-glass paste, Bio-glass powder and placebo paste, Placebo powder and 7% bio-glass paste, Twice a day | Placebo powder and placebo paste | Evaporative, 4-point scale (0, 1, 2, 3) | Test1/test2/test3/control (mean±SD^¶^) |
|  |  |  |  |  |  |  |  | Significance among groups: NR |
|  |  |  |  |  |  |  |  | Evaporative |
|  |  |  |  |  |  |  |  | 1 w: |
|  |  |  |  |  |  |  |  | 0.267±0.458/0.533±0.640/0.8±0.775/1.267±0.594 |
|  |  |  |  |  |  |  |  | 2 w: |
|  |  |  |  |  |  |  |  | 0.133±0.352/0.4±0.632/0.467±0.516/1.067±0.458 |
|  |  |  |  |  |  |  |  | 3 w: |
|  |  |  |  |  |  |  |  | 0.067±0.258/0.133±0.352/0.133±0.352/0.667±0.617 |
|  |  |  |  |  |  |  |  | 6 w: |
|  |  |  |  |  |  |  |  | 0±0/0.067±0.258/0±0/0.067±0.258 |
|  | Milleman JL (2012) [19] | 139 participants | 30-70 yrs (mean 44/43 yrs) | Baseline, Imm, 4 w | NUPRO Sensodyne Prophylaxis Paste with 15% CSPS but without fluoride, Post-prophy | NUPRO Classic without fluoride | Tactile (gram), evaporative, Self-assessed sensitivity 4-point Schiff-score (0, 1, 2, 3) | Test/control (mean±SD) |
|  |  |  |  |  |  |  |  | Tactile |
|  |  |  |  |  |  |  |  | Baseline: 10.56±1.59/10.98±2.27 |
|  |  |  |  |  |  |  |  | Imm: 19.61±11.31*/12.01±5.52 |
|  |  |  |  |  |  |  |  | 4 w: 19.89±11.46*/12.07±5.20 |
|  |  |  |  |  |  |  |  | Evaporative |
|  |  |  |  |  |  |  |  | Baseline: 1.72±0.58/1.65±0.60 |
|  |  |  |  |  |  |  |  | Immediate: 0.88±0.64*/1.72±1.70 |
|  |  |  |  |  |  |  |  | 4 w: 0.87±0.63*/1.67±0.67 |
|  |  |  |  |  |  |  |  | Self-assessed sensitivity |
|  |  |  |  |  |  |  |  | Baseline: 0.82±0.72/0.76±0.74 |
|  |  |  |  |  |  |  |  | Post-scaling: 1.31±0.79/1.28±0.72 |
|  |  |  |  |  |  |  |  | Imm: 1.00±0.90/1.09±0.69 |
|  |  |  |  |  |  |  |  | 4 w: 0.60±0.62/0.72±0.69 |
|  | Li C (2013) [31] | 88 participants | 21-62 yrs (mean 37.4/36.8 yrs) | Baseline, 4 w | Toothpaste containing 5% CSPS (the CSPS concentration was obtained by contacting the company that provided the toothpaste), Twice a day | Toothpaste without CSPS | Evaporative, VAS (0-10 cm) | Test/control (mean±SD) |
|  |  |  |  |  |  |  |  | Evaporative |
|  |  |  |  |  |  |  |  | Baseline: 4.25±1.87/4.03±1.92 |
|  |  |  |  |  |  |  |  | 4 w: 2.53±1.22*/3.63±1.45 |
|  | Neuhaus KW (2013) [33] | 149 participants | 18-70 yrs | Baseline, Imm, 4 w | NUPRO Sensodyne Prophylaxis Paste with 15% CSPS but without fluoride, Post-prophy | NUPRO Classic without CSPS without fluoride | Tactile (gram), evaporative, 4-point scale (0, 1, 2, 3), Self-assessed sensitivity, 4-item verbal description scale (1, 2, 3, 4) | Test1/control (mean±SD) |
|  |  |  |  |  |  |  |  | Tactile |
|  |  |  |  |  |  |  |  | Baseline: 10.38±1.35/10.60±1.64 |
|  |  |  |  |  |  |  |  | Imm: 22.74±12.99*/11.20±3.12 |
|  |  |  |  |  |  |  |  | 4 w: 20.58±11.32*/11.77±5.95 |
|  |  |  |  |  |  |  |  | Evaporative |
|  |  |  |  |  |  |  |  | Baseline: 1.93±0.59/1.97±0.63 |
|  |  |  |  |  |  |  |  | Imm: 1.07±0.62*/1.97±1.70 |
|  |  |  |  |  |  |  |  | 4 w: 0.99±0.55*/2.03±0.67 |
|  |  |  |  |  |  |  |  | Self-assessed sensitivity |
|  |  |  |  |  |  |  |  | Baseline: 0.67±0.68/0.86±0.76 |
|  |  |  |  |  |  |  |  | Post-scaling: 1.19±0.72/1.36±0.75 |
|  |  |  |  |  |  |  |  | Imm: 0.96±0.74/1.20±0.90 |
|  |  |  |  |  |  |  |  | 4 w: 0.52±0.64*/0.88±0.79 |

CSPS: calcium sodium phosphosilicate; PPO: placebo olive oil; RCT: randomized controlled trial; VAS: visual analogue scale; SD: standard deviation; SE: standard error; NR: not reported; ppmF: parts per million fluoride; yrs: years; Imm: immediately; w: weeks; m: months; d: days; h: hours; 95% CIs: 95% confidence intervals; #: extracted from a similar Chinese study and confirmed by contacting the author(s); ^¶^: calculated based on the original data; *: p<0.05 compared with the control group
