# Supplementary material for: The Effect of Calcium Sodium Phosphosilicate on Dentin Hypersensitivity: A Systematic Review and Meta-Analysis
Source: PLoS One. 2015 Nov 6;10(11):e0140176. doi: 10.1371/journal.pone.0140176 (PMC4636152; doi:10.1371/journal.pone.0140176)
Supplement: S4 Table — (DOCX) [file pone.0140176.s005.docx]

**S4 Table. GRADE Profile Table**

**Question: Toothpaste containing 5% CSPS versus a negative control for dentin sensitivity**

**Population:** Subjects with dentin sensitivity

| **Quality assessment** | | | | | | | **No. of patients** | | **Effect** | | **Quality** |
| --- | --- | --- | --- | --- | --- | --- | --- | --- | --- | --- | --- |
|  |  |  |  |  |  |  |  |  |  |  |  |
| **No. of studies** | **Design** | **Risk of bias** | **Inconsistency** | **Indirectness** | **Imprecision** | **Other considerations** | **5% Novamin** | **Negative control; Evaporative, 2 weeks** | **Relative (95% CIs)** | **Absolute** |  |
| **VAS (evaporative, 2 weeks) Better indicated by lower values** | | | | | | | | | | | |
| 4 | Randomized trials | No serious risk of bias | No serious inconsistency | No serious indirectness | Serious^1^ | None | 106 | 107 | (1.15 to 0.20 lower) | MD 0.68 lower | ⊕⊕⊕O MODERATE |
| **VAS (evaporative, 6 weeks) Better indicated by lower values** | | | | | | | | | | | |
| 3 | Randomized trials | No serious risk of bias | No serious inconsistency | No serious indirectness | Serious^1^ | None | 96 | 97 | (1.86 to 1.52 lower) | MD 1.69 lower | ⊕⊕⊕O MODERATE |

^1^ The number of participants from the 4 studies totaled less than 400.

| **Quality assessment** | | | | | | | **No. of patients** | | **Effect** | | **Quality** |
| --- | --- | --- | --- | --- | --- | --- | --- | --- | --- | --- | --- |
|  |  |  |  |  |  |  |  |  |  |  |  |
| **No. of studies** | **Design** | **Risk of bias** | **Inconsistency** | **Indirectness** | **Imprecision** | **Other considerations** | **5% Novamin** | **Negative control; Evaporative; 6 weeks** | **Relative (95% CIs)** | **Absolute** |  |
| **VAS (thermal, 2 weeks) Better indicated by lower values** | | | | | | | | | | | |
| 4 | Randomized trials | No serious risk of bias | No serious inconsistency | No serious indirectness | Serious^1^ | None | 106 | 107 | (1.33 lower to 0.14 higher) | MD 0.59 lower | ⊕⊕⊕O MODERATE |
| **VAS (thermal, 6 weeks) Better indicated by lower values** | | | | | | | | | | | |
| 3 | Randomized trials | No serious risk of bias | No serious inconsistency | No serious indirectness | Serious^1^ | None | 96 | 97 | (2.17 to 1.23 lower) | MD 1.70 lower | ⊕⊕⊕O MODERATE |

^1^ The number of participants from the 3 studies totaled less than 400.

**Question: Prophylaxis paste containing 15% CSPS versus a negative control for dentin sensitivity**

**Population:** Subjects receiving periodontal therapy

| **Quality assessment** | | | | | | | **No. of patients** | | **Effect** | | **Quality** |
| --- | --- | --- | --- | --- | --- | --- | --- | --- | --- | --- | --- |
|  |  |  |  |  |  |  |  |  |  |  |  |
| **No. of studies** | **Design** | **Risk of bias** | **Inconsistency** | **Indirectness** | **Imprecision** | **Other considerations** | **15% Novamin** | **Control, tactile, immediate** | **Relative (95% CIs)** | **Absolute** |  |
| **VAS (evaporative, immediate) Better indicated by lower values** | | | | | | | | | | | |
| 2 | Randomized trials | No serious risk of bias | No serious inconsistency | No serious indirectness | Serious^1^ | Reporting bias^2^ | 97 | 96 | (1.23 to 0.51 lower) | MD 0.87 lower | ⊕⊕OO LOW |
| **VAS (evaporative, 4 weeks) Better indicated by lower values** | | | | | | | | | | | |
| 2 | Randomized trials | No serious risk of bias | No serious inconsistency | No serious indirectness | Serious^1^ | Reporting bias^2^ | 97 | 94 | (1.11 to 0.75 lower) | MD 0.93 lower | ⊕⊕OO LOW |
| **VAS (tactile, immediate) Better indicated by lower values** | | | | | | | | | | | |
| 2 | Randomized trials | No serious risk of bias | No serious inconsistency | No serious indirectness | Serious^1^ | Reporting bias^2^ | 97 | 96 | (12.17 to 7.01 lower) | MD 9.59 lower | ⊕⊕OO LOW |
| **VAS (tactile, 4 weeks) Better indicated by lower values** | | | | | | | | | | | |
| 2 | Randomized trials | No serious risk of bias | No serious inconsistency | No serious indirectness | Serious^1^ | Reporting bias^2^ | 97 | 94 | (10.87 to 5.8 lower) | MD 8.34 lower | ⊕⊕OO LOW |

^1^ The number of participants from the two studies totaled less than 400.
^2^ Both studies were performed by the same group of authors and funded by the same company.
