# Supplementary material for: The Effect of Calcium Sodium Phosphosilicate on Dentin Hypersensitivity: A Systematic Review and Meta-Analysis
Source: PLoS One. 2015 Nov 6;10(11):e0140176. doi: 10.1371/journal.pone.0140176 (PMC4636152; doi:10.1371/journal.pone.0140176)
Supplement: S5 Table — (DOCX) [file pone.0140176.s006.docx]

**S5 Table. Risk of bias assessments in the included studies**

| Du Min Q (2008) [30] | | |
| --- | --- | --- |
| Item | Authors’ judgment | Description |
| Random sequence generation | Yes | Quote: “Subjects demonstrating both air and cold water sensitivity were accepted into the study and randomly assigned to one of the three groups using a computer generated randomization table.” |
| Allocation concealment | Unclear | Comment: Insufficient information to judge |
| Blinding of participants and personnel | Unclear | Comment: Insufficient information regarding the blinding of personnel  Quote: “The study was a longitudinal, double-blind parallel arm study comparing two commercially-available products used for the relief of dentin hypersensitivity.” |
| Blinding of outcome assessment | Yes | Comment: Double blind  Quote: “The study was a longitudinal, double-blind parallel arm study comparing two commercially-available products used for the relief of dentin hypersensitivity.” |
| Incomplete outcome data addressed | Unclear | Comment: Insufficient information regarding the number of and reasons for patients’ failure to follow up in each group  Quote: “A total of four subjects failed to complete the study.” |
| Free of selective reporting | Yes | Comment: Sensitivity scores regarding both evaporative and thermal stimuli were reported for all treatment groups at all time points. |
| Loss to follow up less than 10% | Yes | Quotes: “A total of 75 patients were entered into the study, with 25 in each group.” “A total of four subjects failed to complete the study.” |
| Baseline characteristics balanced | Yes | Comment: No significant difference was found between the Novamin® and placebo groups at baseline. |
| Eligibility criteria specified | Yes | Comment: Inclusion and exclusion criteria were reported. |
| Litkowski L (2010) [32] | | |
| Item | Authors’ judgment | Description |
| Random sequence generation | Unclear | Comment: Insufficient information to judge.  Quote: “Participants meeting all inclusion criteria with no exclusions were randomly assigned to an eight-week treatment schedule of unsupervised brushing twice a day with one of the three dentifrices; placebo, 2.5% NovaMin, or 7.5% NovaMin.” |
| Allocation concealment | Unclear | Comment: Insufficient information to judge.  Quote: “Participants meeting all inclusion criteria with no exclusions were randomly assigned to an eight-week treatment schedule of unsupervised brushing twice a day with one of the three dentifrices; placebo, 2.5% NovaMin, or 7.5% NovaMin.” |
| Blinding of participants and personnel | Unclear | Comment: Insufficient information regarding the blinding of personnel  Quote: “This was a randomized, double-blind, placebo-controlled pilot study.” |
| Blinding of outcome assessment | Yes | Comment: Double blind  Quote: “This was a randomized, double-blind, placebo-controlled pilot study.” |
| Incomplete outcome data addressed | Yes | Quote: “All subjects completed the study.” |
| Free of selective reporting | Yes | Comment: Sensitivity scores to both tactile and thermal stimuli were reported for all treatment groups at all time points. |
| Loss to follow up less than 10% | Yes | Quote: “All subjects completed the study.” |
| Baseline characteristics balanced | Yes | Quote: “No significant differences in the distribution of males and females, distribution of race, mean age at baseline, mean thermal VAS score at baseline, or mean tactile VAS score at baseline were found among the three treatment groups.” |
| Eligibility criteria specified | Yes | Comment: Inclusion and exclusion criteria were reported. |
| Pradeep AR (2010) [14] | | |
| Item | Authors’ judgment | Description |
| Random sequence generation | Yes | Quote: “Selected subjects were randomly assigned to one of three treatment groups by lottery method.” |
| Allocation concealment | Yes | Quote: “The toothpastes were dispensed in tubes labeled A, B, and C, the contents of which were disclosed to the investigators only after completion of the statistical analyses.” |
| Blinding of participants and personnel | Yes | Comment: Triple-masked  Quote: “The study was a single-center, longitudinal, triple masked (investigators, subjects, and statistician), randomized parallel-arm design.” “Investigators (ARP and AS) and patients were masked to toothpaste content.” |
| Blinding of outcome assessment | Yes | Comment: Triple-masked  Quote: “The study was a single-center, longitudinal, triple masked (investigators, subjects, and statistician), randomized parallel-arm design.” “Investigators (ARP and AS) and patients were masked to toothpaste content.” |
| Incomplete outcome data addressed | Yes | Comment: The numbers of participants who failed to follow up in each group were balanced.  Quote: “A total of 120 subjects were included in the study and categorized into three groups, each containing 40 subjects. A total of 110 subjects (58 males and 52 females) were ﬁnally considered because 10 subjects failed to follow up or discontinued the treatment.” “There were 36 subjects in the calcium sodium phosphosilicate group, 37 in the potassium nitrate group, and 37 in the placebo group.” |
| Free of selective reporting | Yes | Comment: Sensitivity scores to both air and water stimuli for all treatment groups at all time points were reported. |
| Lose to follow up less than 10% | Yes | Quote: “A total of 120 subjects were included in the study and categorized into three groups, each containing 40 subjects. A total of 110 subjects (58 males and 52 females) were ﬁnally considered because 10 subjects failed to follow up or discontinued the treatment.” |
| Baseline characteristics balanced | Yes | Quote: “VAS scores for air stimulus of all three groups were not statistically different from each other at baseline.” “VAS scores for water stimulus were not statistically different among the groups at baseline.” |
| Eligibility  criteria specified | Yes | Comment: Inclusion and exclusion criteria were reported. |
| Salian S (2010) [34] | | |
| item | Authors’ judgment | Description |
| Random sequence generation | Yes | Comment: There was insufficient information in the article to judge, so we e-mailed the author and received his reply.  Quote: “This was a single-center, randomized, controlled, double-blind study.” “After the collection of the baseline data, the subjects were randomly divided into three groups of ten subjects each.”  The author’s reply: “The randomization was performed by lottery, where in the names of the enrolled participants were written on a piece of paper and folded. At any point, at least 5 patients' names were included in the lottery. The 3rd investigator then picked up a slip containing a patient's name and created a pre-coded blinded sample.” |
| Allocation concealment | Yes | Comment: There was insufficient information in the article to judge, so we e-mailed the author and received his reply.  Quote: “In order to maintain the double-blind nature of the study, the dentifrices were dispensed by a third party so that neither the examiner nor the patients knew the contents of the toothpaste.”  The author’s reply: “Once all of the patients who met the inclusion criteria were enrolled, a 3rd investigator (who was blinded to the contents of the packets) delivered the samples to the patients. The 1st investigator, who was recording the data, was blinded to the 3rd investigator and the packet that was delivered to the patient…. The allocation sequences were concealed by the lottery system, which was operated by the 3rd blinded investigator who was not involved in data recording.” |
| Blinding of participants and personnel | Yes | Quote: “In order to maintain the double-blind nature of the study, the dentifrices were dispensed by a third party so that neither the examiner nor the patients knew the contents of the toothpaste.” |
| Blinding of outcome assessment | Yes | Comment: Double blind  Quote: “This was a single-center, randomized, controlled, double-blind study.” “In order to maintain the double-blind nature of the study, the dentifrices were dispensed by a third party so that neither the examiner nor the patients knew the contents of the toothpaste.” |
| Incomplete outcome data addressed | Yes | Comment: There was insufficient information in the article to judge, so we e-mailed the author and received his reply.  The author’s reply: “We did not lose any case to follow up.” |
| Free of selective reporting | Yes | Comment: Sensitivity scores to tactile, air and cold water stimuli were reported for all treatment groups at all time points. |
| Loss to follow up less than 10% | Yes | Comment: There was insufficient information in the article to judge, so we e-mailed the author and received his reply.  The author’s reply: “We did not lose any patient to follow up.” |
| Baseline characteristics balanced | Yes | Quote: “Since the baseline scores for all three groups were similar and did not show any significant differences” |
| Eligibility criteria specified | Yes | Comment: Inclusion and exclusion criteria were reported. |
| Pradeep AR (2012) [20] | | |
| Item | Authors’ judgment | Description |
| Random sequence generation | Yes | Quote: “A total of 160 subjects were included in the study, randomized and categorized into four groups, each containing 40 subjects. The randomization was performed using a computer-generated random table.” |
| Allocation concealment | Yes | Quote: “Investigators were neither involved in the randomization process nor were they aware of the assigned groups.” |
| Blinding of participants and personnel | Yes | Comment: Triple-masked  Quote: “The study was a single centre, longitudinal, triple masked (investigators, subjects and statistician), randomized parallel-arm design.” |
| Blinding of outcome assessment | Yes | Comment: Triple-masked  Quote: “The study was a single centre, longitudinal, triple masked (investigators, subjects and statistician), randomized parallel-arm design.” “The toothpastes were dispensed in white tubes by the investigator (ARP) labelled A, B, C and D, the contents of which were disclosed to the investigators only after completion of the statistical analyses.” |
| Incomplete outcome data addressed | Yes | Quote: “A total of 160 subjects were included in the study, randomized and categorized into four groups, each containing 40 subjects.” “There were 39 subjects in Group 1, 36 in Group 2, 38 in Group 3 and 37 in Group 4 who ﬁnally completed the study.” |
| Free of selective reporting | Yes | Comment: Sensitivity scores to both air and water stimuli were reported for all treatment groups at all time points. |
| Lose to follow up less than 10% | Yes | Quote: “A total of 160 subjects were included in the study.” “A total of 149 subjects (72 males and 77 females) were ﬁnally considered because 11 subjects failed to follow up or discontinued the treatment.” |
| Baseline characteristics balanced | Yes | Quote: “No signiﬁcant difference between groups at baseline was found for both air and water stimulus.” |
| Eligibility criteria specified | Yes | Comment: Inclusion and exclusion criteria were reported. |
| GSK clinical trial (2013) [28] | | |
| Random sequence generation | Unclear | Comment: Insufficient information to judge  Quote: “Allocation: Randomized” |
| Allocation concealment | Unclear | Comment: Insufficient information to judge  Quote: “Allocation: Randomized” |
| Blinding of participants and personnel | No | Quote: “Masking: Single Blind (Outcomes assessor)” |
| Blinding of outcome assessment | Yes | Quote: “Masking: Single Blind (Outcomes assessor)” |
| Incomplete outcome data addressed | Yes | Comment: The number of and reasons for participants not completing the study were reported. |
| Free of selective reporting | Yes | Comment: Sensitivity scores to both evaporative and tactile stimuli were reported for all treatment groups at all time points. |
| Loss to follow up less than 10% | Yes | Comment: One of 195 participants did not complete the study according to the information provided. |
| Baseline characteristics balanced | Unclear | Comment: Insufficient information to judge |
| Eligibility criteria specified | Yes | Comment: Inclusion and exclusion criteria were reported. |
| GSK clinical trial (2014) [29] | | |
| Random sequence generation | Unclear | Comment: Insufficient information to judge  Quote: “Allocation: Randomized” “This will be a single center, eight week, randomized, controlled, examiner blind, four treatment arm, parallel design, stratified, exploratory study.” |
| Allocation concealment | Unclear | Comment: Insufficient information to judge  Quote: “Allocation: Randomized” |
| Blinding of participants and personnel | Unclear | Comment: Insufficient information about the blinding of personnel  Quote: “Masking: Double Blind (Subject, Investigator)” |
| Blinding of outcome assessment | Yes | Comment: Double blind  Quote: “Masking: Double Blind (Subject, Investigator)” “This will be a single center, eight week, randomized, controlled, examiner blind, four treatment arm, parallel design, stratified, exploratory study.” |
| Incomplete outcome data addressed | Yes | Comment: All participants completed the study according to the results. |
| Free of selective reporting | Yes | Comment: Sensitivity scores to both evaporative and tactile stimuli were reported for all treatment groups at all time points. |
| Loss to follow up less than 10% | Yes | Comment: All participants completed the study according to the results. |
| Baseline characteristics balanced | Unclear | Comment: Insufficient information to judge |
| Eligibility criteria specified | Yes | Comment: Inclusion and exclusion criteria were reported. |
| Yu X (2011) [35] | | |
| Item | Authors’ judgment | Description |
| Random sequence generation | Yes | Quote: “依随机数字表将患者分为粉膏组(生物玻璃粉剂与生物玻璃膏剂)、粉剂组 (生物玻璃粉剂与安慰膏剂)、膏剂组(安慰粉剂与生物玻璃膏剂)和对照组(粉剂与膏剂均为安慰剂)。” |
| Allocation concealment | Unclear | Quote: “由1名不参与操作和检查的研究者负责分组及实验材料的发放。在实验结束前,所有患者与临床操作者均不了解分组和实验材料的发放情况。” |
| Blinding of participants and personnel | Unclear | Comment: Double blind  Quote: “由1名不参与操作和检查的研究者负责分组及实验材料的发放。在实验结束前,所有患者与临床操作者均不了解分组和实验材料的发放情况。” |
| Blinding of outcome assessment | Yes | Comment: Double blind  Quote: “由1名不参与操作和检查的研究者负责分组及实验材料的发放。在实验结束前,所有患者与临床操作者均不了解分组和实验材料的发放情况。” |
| Incomplete outcome data addressed | Yes | Comment: There were no missing data according to the results. |
| Free of selective reporting | Yes | Comment: Sensitivity scores to cold stimulus were reported for all treatment groups at all time points. |
| Loss to follow up less than 10% | Yes | Comment: There were no missing data according to the results. |
| Baseline characteristics balanced | Unclear | Comment: A comparison of the sensitivity scores to cold stimulus at baseline was not reported.  Quote: “各组基线PD、CAL及BI均有较好的一致性。” |
| Eligibility criteria specified | Yes | Comment: Inclusion and exclusion criteria were reported. |
| Milleman JL (2012) [19] | | |
| Item | Authors’ judgment | Description |
| Random sequence generation | Yes | Quote: “Excel software was used for randomization. The function =RAND() which returns a random number was used in Column I to produce 300 random numbers. In Column II the letters A, B and C were placed in groups of three, 100 times (i.e., A, B, C, A, B, C etc.). The cells were then blocked in groups of three and sorted by the number in Column I from smallest to largest, which randomized the letters next to those numbers. Assigning this randomization in blocks of three ensured that the groups were evenly distributed.” |
| Allocation concealment | Yes | Comment: Insufficient information to judge  Quote: “The random allocation sequence was generated by the sponsor and a randomization sheet was given to the study site.” “Only the sponsor was aware of the randomization code. The code was not broken during the course of this trial.” |
| Blinding of participants and personnel | Yes | Quote: “All staff members at the study site and all subjects were blinded.” “The details of the groups were unknown to the examiner, the study staff, and the subjects. The statistician was also blinded to the identity of the groups.” |
| Blinding of outcome assessment | Yes | Quote: “All staff members at the study site and all subjects were blinded. The examiner was not present when the prophylaxis procedures and paste application occurred.” |
| Incomplete outcome data addressed | Yes | Comment: No participant was lost to follow up after the treatments began.  Quote: “139 subjects are stratified into treatment groups based on Schiff score…. 139 subjects receive sensitivity questionnaire and measurement by Air and Tactile methods at the 28 day follow-up.” (Figure. Participant flow throughout study) |
| Free of selective reporting | Yes | Comment: Sensitivity scores to tactile and air stimuli and overall whole-mouth pain without outside stimuli for all treatment groups at all time points were reported. |
| Loss to follow up less than 10% | Yes | Comment: No participant was lost to follow up after the treatments began.  Quote: “139 subjects are stratified into treatment groups based on Schiff score.” “139 subjects receive sensitivity questionnaire and measurement by Air and Tactile methods at the 28 day follow-up.” |
| Baseline characteristics balanced | Yes | Comment: No significant differences were observed between treatment Groups A (Novamin without fluoride) and C (control). |
| Eligibility criteria specified | Yes | Comment: Inclusion and exclusion criteria were reported. |
| Li C (2013) [31] | | |
| Item | Authors’ judgment | Description |
| Random sequence generation | Yes | Quote: “随机分组方法：将96例纳入试验的患者从1-96编号， 在计算接上产生 96个随机数，前48个对应试验组，后48个对应对照组，按随机数的大小进行排序，随机数的秩序就是患者的编号，再按患者的编号从小到大排列，对应的组别即为分组方案.” |
| Allocation concealment | Unclear | Comment: Insufficient information to judge |
| Blinding of participants and personnel | Unclear | Comment: Insufficient information about the blinding of personnel  Quote: “遵照随机、双盲、对照的原则” |
| Blinding of outcome assessment | Yes | Comment: Double blind  Quote: “遵照随机、双盲、对照的原则” |
| Incomplete outcome data addressed | Yes | Comment: The numbers of participants who did not complete the study in the intervention and control groups were balanced.  Quote: “按照纳入和排除标准共纳入96例参加试验，最后88完成试验进入结果分析，其余8例未能完成试验的原因与牙膏无关.” “试验组（n=48）,失访（n=4），纳入分析（n=44）” “对照组（n=48）,失访（n=4），纳入分析 (n=44)” (Figure 1) |
| Free of selective reporting | Yes | Comment: Sensitivity scores to the air stimulus were reported for both treatment groups at all time points. |
| Loss to follow up less than 10% | Yes | Quote: “按照纳入和排除标准共纳入96例参加试验，最后88完成试验进入结果分析，其余8例未能完成试验的原因与牙膏无关.” |
| Baseline characteristics balanced | Yes | Quote: “基线时，两组目测类比疼痛评分比较差异无显著性意义(P > 0.05)。” |
| Eligibility criteria specified | Yes | Comment: Inclusion and exclusion criteria were reported. |
| Neuhaus KW (2013) [33] | | |
| Item | Authors’ judgment | Description |
| Random sequence generation | Yes | Quote: “Excel software (Microsoft, Redmond, VA, USA) was used for randomization. The function = RAND() was used in column A for 300 random numbers. In column B, the letters A, B and C were put in groups of 3, 100 times (i.e. A, B, C, A, B, C). The cells were then blocked in groups of 3 and randomized by the random number in column A from smallest number to the largest number. Doing it in blocks of 3 ensured that the groups were evenly distributed.” |
| Allocation concealment | Yes | Quote: “The random allocation sequence was generated by K.A.M. and given  to Salus Research before the start of the study. Salus Research enrolled the patients and the patients were assigned to the specific group numbers.” |
| Blinding of participants and personnel | Yes | Quote: “The entire study was blinded. The prophylaxis paste cups used had silver/blank lidstock and were only identified by a letter on the lidstock. The groups were not known by the examiners or patients. The examiner was in a different section of the building and the study coordinator gave the paste to the hygienist in yet another location of the building…. The aim of this single-site, randomized, controlled, double-blind, 3-arm parallel study was to determine the effectiveness of a prophylaxis paste containing 15% calcium sodium phosphosilicate.” |
| Blinding of outcome assessment | Yes | Comment: Double blind  Quote: “The aim of this single-site, randomized, controlled, double-blind, 3-arm parallel study was to determine the effectiveness of a prophylaxis paste containing 15% calcium sodium phosphosilicate.” |
| Incomplete outcome data addressed | Yes | Comment: The numbers of and reasons for participants not completing the study were reported.  Quote: “A total of 2 subjects were lost following the baseline visit, both due to scheduling conflicts.” |
| Free of selective reporting | Yes | Comment: Sensitivity scores to tactile and air stimuli, as well as self-assessed sensitivity scores, were reported for all treatment groups at all time points. |
| Loss to follow up less than 10% | Yes | Quote: “At the Day 28 examination, 149 evaluable subjects completed the study. A total of 2 subjects were lost following the baseline visit.” |
| Baseline characteristics balanced | Yes | Quote: “All groups were evenly balanced with no statistically significant differences for the baseline values.” |
| Eligibility criteria specified | Yes | Comment: Inclusion and exclusion criteria were reported. |
